# Effect of Tranexamic Acid on Hematologic Values and Blood Loss in Reverse Total Shoulder Arthroplasty

**DOI:** 10.1155/2017/9590803

**Published:** 2017-07-27

**Authors:** Sae Hoon Kim, Whan Ik Jung, Young Jun Kim, Do Hyeon Hwang, Young Eun Choi

**Affiliations:** ^1^Department of Orthopaedic Surgery, Seoul National University Hospital, Seoul, Republic of Korea; ^2^Department of Orthopaedic Surgery, Seoul National University College of Medicine, Seoul, Republic of Korea

## Abstract

**Purpose:**

Use of tranexamic acid (TXA) in the setting of arthroplasty of the lower extremity has been previously described. The aim of this study was to evaluate the benefit of a single dose of TXA (500 mg vial) administered intravenously just prior to RTSA in an Asian population.

**Methods:**

The records of 48 patients (no TXA, *n* = 24, versus TXA, *n* = 24) that underwent RTSA for cuff tear arthropathy were retrospectively reviewed. All patients had a Hemovac drain positioned for 2 days after surgery. Hemoglobin (Hb) and hematocrit (Hct) were checked on postoperative day 2 and compared with preoperative levels.

**Results:**

Hematologic change on postoperative day 2 as determined by Hb level after surgery was statistically lower in the TXA group (2.8 ± 0.8 versus 2.1 ± 0.8 (mg/dL), *P* = 0.006). Mean fall in Hct level was also significantly less in the TXA group (8.0 ± 2.5 versus 6.1 ± 2.6 (L/L), *P* = 0.012). Total Hemovac drainage tended to be lower in the TXA group (263.4 ± 129.3 versus 203.5 ± 84.2 (ml), *P* = 0.064). TXA was found to have no noticeable side effects.

**Conclusion:**

The use of a single intravenous dose of TXA immediately prior to RTSA reduces hematologic deterioration postoperatively and the amount of Hemovac drainage. TXA could avoid unnecessary transfusion and its associated medical side effects and cost.

## 1. Introduction

Since its introduction in the late 1950s, arthroplasty of the shoulder has offered the prospect of improved function and pain relief when native glenohumeral articulation has been damaged by infection, arthritis, or trauma. After that reverse total shoulder arthroplasty (RTSA) was introduced successfully to minimize pain and maximize function for patients with a rotator cuff-deficient shoulder, and its indications are expanding [1].

Like arthroplasties of other joints, shoulder arthroplasty is associated with significant blood loss, sometimes to the extent of requiring blood transfusion. Overall rates of blood transfusion after anatomical TSA have been reported to range from 7.4% to 43% [2–4]. Moreover, due to morphological characteristics and the tendency to create more dead space, RTSA is associated with elevated risks of postoperative blood loss and postoperative hematoma, which has a reported incidence ranging from 1% to 20% [5–7].

Therefore, blood transfusion is sometime necessary after RTSA but can lead to adverse immunologic reactions, disease transmission, intravascular hemolysis, transfusion-related lung injury, renal impairment/failure, and increased medical costs [8, 9]. For these reasons, more effective, safer methods of reducing blood loss and blood transfusion requirements during and after RTSA are needed.

Tranexamic acid (TXA) has been used successfully in other surgical fields and numerous studies on total knee arthroplasty (TKA) and total hip arthroplasty (THA) have explored the efficacies and safeties of intravenous (IV) or topical TXA with respect to reducing blood loss and transfusion rates [10–15]. However, the benefits of TXA administration have not been comprehensively investigated in RTSA. A few comparative studies have been conducted in Caucasian populations on the use of TXA for reducing blood loss after TSA and RTSA [16–18]. However, it is not still clear in Asians with RTSA. Thus, the aim of the present study was to evaluate the benefits of IV TXA in RTSA in an Asian population by comparing the hemoglobin (Hb), hematocrit (Hct), and blood drainage levels after RTSA with or without the prior IV administration of TXA.

## 2. Materials and Methods

### 2.1. Study Design

This retrospective review was conducted using prospectively collected data. All protocols were approved by the Institutional Review Board of our institution (IRB number: H-1607-179-779). From April 2015, TXA has been routinely in patients treated by RTSA at our institute. The present study was conducted by reviewing the archive records of patients treated before (the no TXA group) or after (the TXA group) the adoption of TXA. RTSAs for cuff tear arthropathy were selected to remove confounders, and, thus, revision and tumor reconstruction cases, sequelae after septic arthritis, and fracture sequelae were excluded. In addition, patients that received a transfusion intraoperatively by an anesthesiologist were excluded from the analysis. Patients administered anticoagulant or antiplatelet agents before RTSA were included, but they were instructed to stop taking these medications for at least seven days before surgery, when this was considered safe. Finally, 48 patients (24 patients per group) that underwent RTSA for the treatment of cuff tear arthropathy were chosen in retrograde consecutive fashion from the institutional data base (Table 1). Baseline characteristics were not different in both groups (all *P*'s > 0.05). TXA (500 mg vial) was administered intravenously one hour before surgery. No topical application of TXA was performed.

### 2.2. Surgical Technique

All surgical procedures were performed by a single surgeon (K. S. H.). Despite surgeries being conducted at different times in the two study groups the surgical techniques used were almost identical. All operations were performed through a deltopectoral approach in the beach chair position without the use of a pneumatic arm holder. Briefly, the subscapularis was elevated subperiosteally to achieve enough length for reinsertion and the remaining long head of the biceps was tenotomized and tenodesized at the pectoralis major level. Neck cutting of the humerus was done in a 20° retrograde fashion using an intramedullary version guide. Glenoid exposure and capsular release were performed in a standard manner. The glenoid baseplate and glenosphere were implanted with 3-4 mm of inferior translation and 10° of inferior tilt. For implantation of the humeral component, a cementless stem was used preferably, but cement was used in patient with less favorable bone quality. Subscapularis repair was performed if, but when far retracted, the subscapularis was left unattached to prevent creating too tight a joint. Stability and range of motion were checked, and, before wound closure, a Hemovac drain was inserted at the joint. The deltopectoral interval was closed with an absorbable suture. In all cases the operative shoulder was immobilized using an abduction brace during the entire monitoring period.

### 2.3. Hematologic Tests and Hemovac Drainage

Hb concentration, Hct, and drained blood volume were measured from the day before surgery and on the 1st and 2nd days after surgery. Drainage was checked every morning and Hemovacs were removed on the 2nd day. The total drain output was defined as the sum of recorded drainage outputs.

### 2.4. Statistical Analyses

Statistical analysis was performed using the SPSS software package (version 21.0, IBM SPSS statistics, Chicago, IL). Student's *t-*test was used to evaluate the null hypothesis that mean values in the two study groups were no different for normally distributed data. Two-sided *P* values of <0.05 were considered statistically significant.

## 3. Results

Hb and Hct levels were significantly lower in the TXA and no TXA groups at 2 days postoperatively (TXA group: 13.4 ± 1.1 mg/L and 40.1 ± 3.6 L/L preoperatively and 11.3 ± 1.2 mg/L and 34.0 ± 4.2 L/L postoperatively and no TXA group: 13.0 ± 1.2 mg/L and 38.5 ± 3.3 L/L preoperatively and 10.2 ± 1.1 mg/L and 30.5 ± 3.2 L/L, postoperatively, resp.) (Table 2), and the reductions in Hb and Hct levels were significantly greater in the no TXA group. Mean blood drainage amount during the first 2 days tended to be greater in the no TXA group (*P* = 0.064). The use of TXA was not found to be associated with any notable side effect. Subgroup analysis showed implant type, humeral component cementing, and subscapularis repair had no effects on Hb and Hct levels, falls in Hb and Hct levels, or amount of Hemovac drainage (all *P* > 0.05). Included cohorts did not show any notable complication in 1-year follow-up and postoperative ASES scores were measured as 76.1 ± 10.3 in no TXA group and 81.4 ± 12.3 in TXA group which showed no statistical difference in both groups (*P* = 0.271).

## 4. Discussion

Although the number of cases was small, hematologic data were statistically better after the administration of IV TXA prior to RTSA in an Asian population. The amount of Hemovac drainage tended to be less in the TXA group. The side effects of TXA are pale skin, nausea, vomiting, and color vision problems, but none of these complications was encountered in the present study. In view of the low cost of TXA (~30 cents/vial, 500 mg) and its safety, it would appear to be suitable for use in surgeries raising concerns of blood loss, especially during arthroplasty surgery, which is associated with bone bleeding problems achieving hemostasis.

TXA acts as an antifibrinolytic that competitively inhibits the activation of plasminogen and thereby reduces the conversion of plasminogen into plasmin. In addition, at higher doses, TXA also directly inhibits plasmin activity [19] and may improve platelet function in patients administered dual antiplatelet therapy [20].

Recently, the use of TXA has been described during shoulder arthroplasty in Caucasian population [16–18]. Therefore, we considered that an evaluation of its effectiveness in an Asian population would be meaningful, because race may affect the coagulation system; for example, racial differences have been detected in the prevalence of deep vein thrombosis [21, 22]. The reasons for these differences are unclear but may be due to true biologic differences, differences in the prevalence of comorbidities leading to venous thromboembolism, or results of complications among venous thromboembolism patients [23]. Moreover, warfarin dose requirements have previously been shown to be greater for African Americans than for Asians or Caucasians [24], because of racial differences in genotype frequencies [25].

Gillespie et al. [18] reported on the similar effectiveness of topical TXA (100 mL of normal saline with 2 g TXA) on reducing blood loss after RTSA and TSA and evaluated the effect of topical TXA on Hemovac drain output after shoulder arthroplasty. Like that observed in the present study, topical TXA decreased drain output after TSA and RTSA (by 100 and 50 mL, resp.). Abildgaard et al. [16] retrospectively analyzed the effect of IV TXA (1 g) after TSA and RTSA and found that TXA diminished Hb and Hct reductions drain outputs postoperatively. In a retrospective study, Friedman et al. [17] found IV TXA (20 mg/kg) effectively reduced Hb and Hct level changes and hospital stays after TSA and RTSA. RTSA differs substantially from TSA in terms of implanting glenoid and humeral components, and its reverse configuration design and the absence of the cuff tendon create more dead space. TXA has been used for some time for arthroplasties of the hip and knee, and published results have allowed systematic reviews and meta-analyses to be undertaken [26–28]. These studies concluded IV and intra-articular TXA are safe and effective in reducing blood loss and blood transfusion requirements but that they do not increase the risk of postoperative DVT. The postoperative blood-saving effect of TXA after TKA has been previously reported to be between 20% and 48%, that is, equivalent to as much as 833 mL [13, 15, 29]. It has also been suggested that topical administration may be superior to the intravenous route [16]. However, further research is required to determine the optimum route or dose for administration.

In the present study, no patient received a blood transfusion. Patients that received a transfusion intraoperatively by an anesthesiologist were excluded from the analysis. Decisions regarding intraoperative transfusions were made by the responsible anesthesiologist and were based on considerations of patient age, medical history, extent of the surgery, surgical time, and intraoperative hematocrit level without any absolute guideline. Since no consensus has been reached regarding transfusion and the subjective nature of the decisions, we decided to exclude eight cases that received a transfusion intraoperatively, that is, 3 cases in the no TXA group and 5 in the TXA group.

Patients were asked not to take antiplatelet medications for at least 7 days before surgery. There were 14 cases of taking at least aspirin in the study. Furthermore, subanalysis showed cement usage for humeral component fixation and subscapularis repair did not significantly affect Hb and Hct levels or blood loss.

The present study has several limitations. First, the sample size was small, nevertheless significant results were obtained, and focus on RTSA increased statistical power because hemodynamic changes are more pronounced after RTSA compared to TSA. Second, the study is inherently limited by its retrospective design and the use of a historical control group. Nevertheless, pre- and post-op protocols were identical and all surgeries were conducted by the same surgeon. Accordingly, we do not believe the difference between group treatment times introduced bias.

## 5. Conclusions

The administration of a single dose of TXA intravenously prior to RTSA reduced hematologic deterioration postoperatively and Hemovac drainage. The study shows the use of TXA could avoid unnecessary transfusions and their associated side effects and costs.

## Figures and Tables

**Table 1:** Baseline data of the no TXA and TXA groups.

	No TXA (24)	TXA (24)	*P* value
Age (yr)	74.2 ± 4.4	73.2 ± 4.4	0.435
Sex (M : F)	6 : 18	3 : 21	0.461
Body mass index	25.1 ± 3.7	25.0 ± 3.4	0.896
Antiplatelet drug medication	8	6	0.525
American Shoulder and Elbow Surgeon score	41.3 ± 18.4	39.0 ± 18.2	0.679
Implant (A : B : C)	12 : 9 : 3	13 : 7 : 4	0.805
Cementing of humeral component	5	2	0.416
Subscapularis repair	19	21	0.701
Operation time (min)	66.1 ± 8.2	62.9 ± 11.9	0.282
Preop Hb (mg/L)	13.0 ± 1.2	13.4 ± 1.1	0.239
Preop Hct (L/L)	38.5 ± 3.3	40.1 ± 3.6	0.110

TXA = trenexamic acid; M = male; F = female; Preop = preoperative; Hb = hemoglobin; Hct = hematocrit; Data are presented as means ± standard deviations; Implant A = DJO Reverse Shoulder Prosthesis (DJO, Austin, TX, USA), B = Tornier reverse shoulder prosthesis (Tornier Inc, Stafford, TX, USA), C = Biomet Comprehensive Reverse Total Shoulder Replacement (Biomet Inc, Warsaw, IN, USA).

**Table 2:** Postoperative hematologic variables and blood drainage.

	No TXA (24)	TXA (24)	*P* value
Post-op Hb (mg/dL)	10.2 ± 1.1	11.3 ± 1.2	0.003
Drop of Hb (mg/dL)	2.8 ± 0.8	2.1 ± 0.8	0.006
Post-op Hct (L/L)	30.5 ± 3.2	34.0 ± 4.2	0.002
Drop of Hct (L/L)	8.0 ± 2.5	6.1 ± 2.6	0.012
Total Hemovac drain (ml)	263.4 ± 129.3	203.5 ± 84.2	0.064

TXA = tranexamic acid; Hb = hemoglobin; Hct = hematocrit; Data were presented as mean ± standard deviation.
